# Behavior of medial gastrocnemius muscle beneath kinesio taping during isometric contraction and badminton lunge performance after fatigue induction

**DOI:** 10.1038/s41598-023-28818-3

**Published:** 2023-01-31

**Authors:** Minh Hoang-Thuc Vo, Chien-Ju Lin, Hsiao-Feng Chieh, Li-Chieh Kuo, Kai-Nan An, Yu-Lin Wang, Fong-Chin Su

**Affiliations:** 1 Department of Biomedical Engineering, National Cheng Kung University, Tainan, Taiwan; 2 Department of Occupational Therapy, National Cheng Kung University, Tainan, Taiwan; 3 Division of Orthopedic Research, Mayo Clinic, Rochester, USA; 4 Department of Rehabilitation, Chi Mei Medical Center, Tainan, Taiwan

**Keywords:** Biomedical engineering, Rehabilitation, Fatigue, Ultrasound

## Abstract

Kinesio taping (KT) is widely used in sports for performance improvement and injury prevention. However, little is known of the behavior of the muscle region beneath the KT with movement, particularly when the muscle is fatigued. Accordingly, this study investigated the changes in the medial gastrocnemius muscle architecture and fascia thickness when using KT during maximum isometric plantar flexion (MVIC) and badminton lunges following heel rise exercises performed to exhaustion. Eleven healthy collegiate badminton players (4 males and 7 females) were recruited. All of the participants performed two tasks (MVIC and badminton lunge) with a randomized sequence of no taping, KT and sham taping and repeated following exhaustive repetitive heel rise exercise. In the MVIC task, the fascia thickness with the medial gastrocnemius muscle at rest significantly decreased following fatigue induction both without taping and with KT and sham taping (*p* = 0.036, *p* = 0.028 and *p* = 0.025, respectively). In the lunge task, the fascia thickness reduced after fatigue induction in the no taping and sham taping trials; however, no significant change in the fascia thickness occurred in the KT trials. Overall, the results indicate that KT provides a better effect during dynamic movement than in isometric contraction.

## Introduction

As one of the world's fastest racket sports^1^, badminton requires players to reach the shuttlecock as fast as possible and with the minimal effort within the physical constraints of the court^2^. In order to meet this requirement, badminton players must master a range of specific footwork strategies. Among these strategies, one of the most important is the forward lunge, which accounts for up to 17.86 ± 4.83% of the movements performed during a singles match^3^. Due to the rapid, repetitive, and stop-and-go nature of the lunge movement, the leg muscles may easily become fatigued and painful, particularly in long rallies. Fatigued muscles not only harm athletic performance, but also increase the risk of injury^4^. Thus, athletes (including badminton players) commonly apply kinesio tape (KT) to the muscle region after suffering muscle fatigue during training or competition^5,6^.

**Figure 1:**
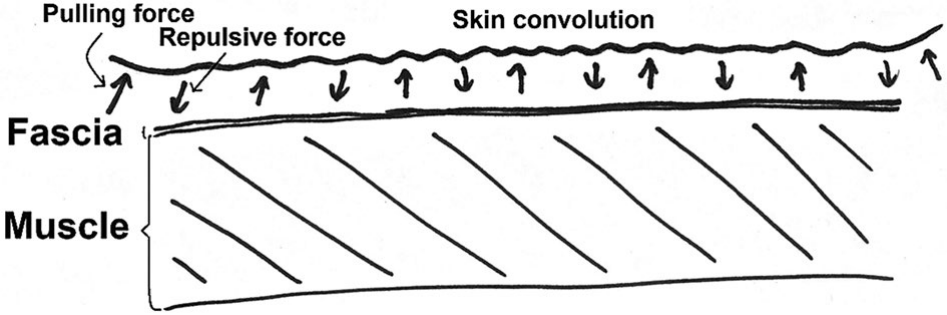
Impact of KT on structure beneath skin.

According to the inventor of KT, Dr. Kenzo Kase’s report, KT exerts five main physiological effects: skin, circulatory/lymphatic, fascia, muscle, and joint^7^, where these effects are induced through a recoil action produced by the wave-pattern elastic nature of the tape, which leads to skin convolutions of the involved region. For example, by lifting the skin, the tape creates space in the muscle fascia—a soft connective tissue wrapping the muscle fibers, which contains nerves, blood and lymphatic vessels^8,9^. The study in rabbit hind leg by Shim et al. resulted that the tape induces lymphatic drainage during passive exercise and hastened blood flow to the taped area^10^, thereby helping reduce swelling and speeding up the self-healing process. Thus, as described above, athletes often make use of adjuncts such as braces and taping techniques to speed the muscle recovery process following intensive training or tournament play^11^. Many studies have examined the effects of KT on ability enhancement in vertical jumping^12^, or in improving the medial gastrocnemius muscle strength and push-off force in healthy inactive individuals^13^. It has been shown that KT provides an effective means of enhancing the recovery from delayed onset muscle soreness by elevating the skin and thus improving the muscle oxygenation^14–16^. KT has also been shown to be highly effective in improving the performance of countermovement jumping tasks^17^ and in alleviating the effects of muscle fatigue^18,19^ when applied to the gastrocnemius muscle in healthy subjects. Pamuk and Yucesoy^20^ used a magnetic resonance imaging (MRI) technique to investigate the effects of KT on the fascia and tibialis anterior muscle region beneath the tape. Wang et al.^21^ showed that the magnetic resonance elastography-derived shear stiffness of the lumbar paraspinal muscles reduced following the application of KT. Cimino et al.^22^ found that KT both thickened and attenuated the skin along the edges on the lower back depending on the spine posture^22^. It was thus inferred that the KT simultaneously produced both a pulling force and a repulsive force on the skin, as shown in Fig. 1.

However, while the studies above provide useful insights into the effects of KT on muscles at rest, the literature contains very little information on the acute effects of KT under dynamic movement. It has been suggested that the constant stretching and recoiling of KT during movement may mimic the effects of myofascial release in increasing blood flow to the injured area and improving lymphatic drainage by eliciting sustained pressure^23^. Accordingly, in the present study, it is hypothesized that KT recoil applies force on the epimysium of the underlying muscle and releases the fatigue-induced tightened thinner fascia layer^24^, thereby increasing its thickness. It is further hypothesized that if the recoil-induced force reaches as far as the endomysium, i.e., form of fascia ensheathing each individual muscle fiber^8^, it may prompt an alteration of the underlying muscle architecture, thereby changing the muscle contraction behavior^25^. To test these hypotheses, an ultrasound technique is employed to observe the pennation angle, fascicle length and fascia thickness of the medial gastrocnemius muscle beneath KT in collegiate badminton players performing maximum isometric plantar flexion (MVIC) and badminton lunges before and after heel rise exercise performed to exhaustion, respectively (Supplementary Figures S1–S4).

## Materials and methods

### Participants

Eleven right-handed players (4 males and 7 females) had an average age of 21.2 ± 1 years old, an average height of 166.5 ± 8.6 cm, and an average weight of 62.2 ± 6.4 kg from National Cheng Kung University’s badminton team voluntarily participated in the study. None of the participants had a history of heart disease, asthma, neurological impairment, musculoskeletal injuries within the previous month, or allergic reactions to KT, adhesive tape, or ultrasound gel. The experiments were approved by the Institutional Review Board of National Cheng Kung University. All methods were performed in accordance with the relevant guidelines and regulations.

### Participant involvement

Participants were involved in the conduct of this research. Participants first noticed the recruitment poster at the school badminton court and contact for consulting the eligibility and made an appointment at their available time. All of the participants were instructed the experiment in detail and signed on the committee-approved informed consent before taking part in the study. They had the rights to withdraw the experiment at any time if they felt burden of the intervention and time required.

### Experimental design

#### Taping application

**Figure 2:**
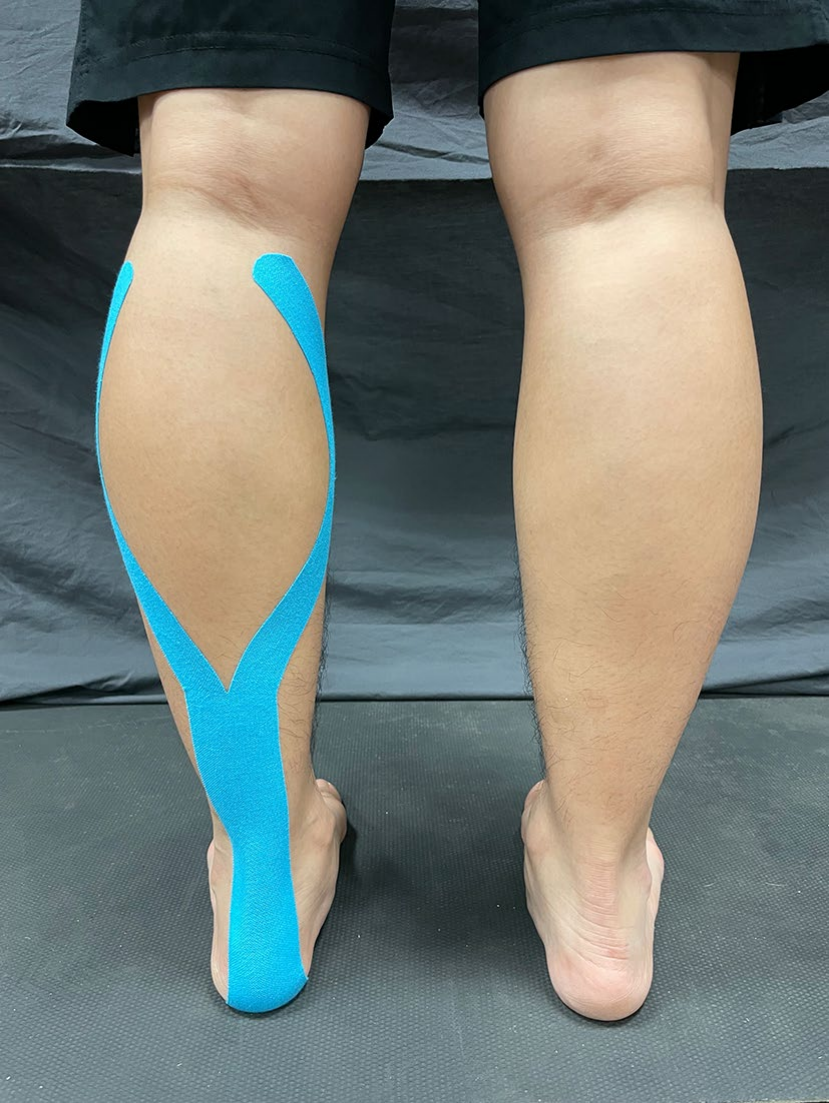
The Y-shaped taping technique.

Two kinds of tape were randomly applied to the participants, namely elastic adhesive tape (3M™ Health Care, Taiwan) as sham taping and kinesio tape (NITTO Medical Kinesiology Tape NK-50, Japan) as elastic therapeutic taping. For both tapes, a Y-shape taping technique was employed to induce activation of the gastrocnemius muscle through the application of proximal to distal tension as demonstrated in Fig. 2. The tape was applied by the same trained practitioner in every case. Briefly, Y-shaped KT (or sham taping) was applied to the gastrocnemius muscle of the left leg with 30% tension over the muscle. To achieve the required taping tension, the original length of the tape was measured with the foot placed in a relaxed, prone position with the knee fully extended and the feet on an examination table. The measured length was then multiplied by 0.7 to obtain the practical length of the tape. The distal I-shaped head of the tape was applied from the calcaneus along the soleus muscle up to the gastrocnemius muscle, while the two proximal Y-shaped heads were attached over the margins of the medial and lateral heads of the gastrocnemius muscle, respectively.

#### Fatigue protocol

**Figure 3:**
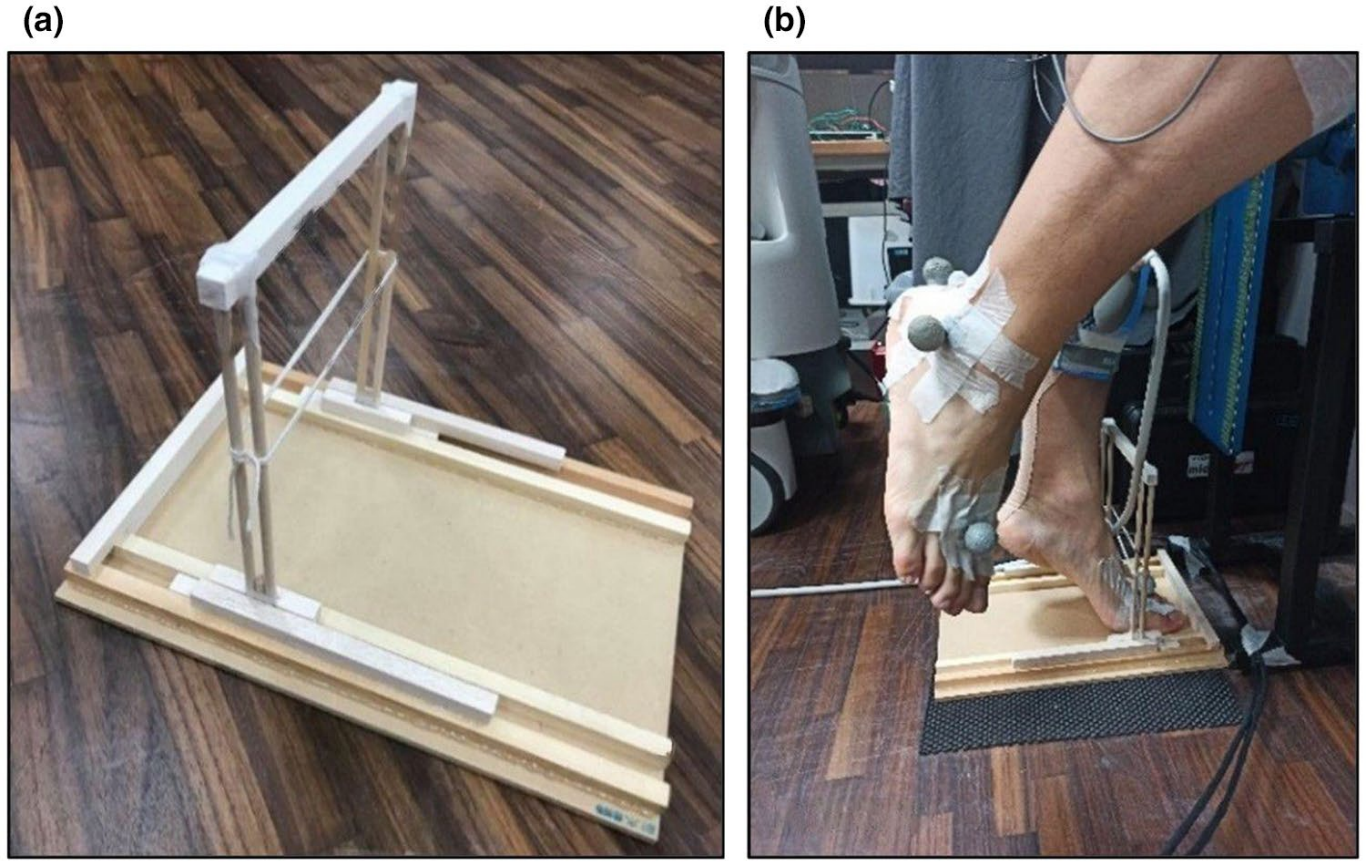
Custom-built Haberometer (**a**) and single heel rise (**b**).

The fatigue protocol was performed with the non-dominant leg placed on a custom-made Haberometer^26^ (see Fig. 3a). A horizontal string was adjusted to the height of the navicular bone of each subject at their maximal heel rise height. During the fatigue protocol, the examined leg was fully extended, the contralateral leg was suspended in the air, and three fingertips of one hand were placed on a wall for balance. Each subject raised his/her heel until the navicular bone touched the string and then lowered it back to the mat, as shown in Fig. 3b. The protocol was performed at a speed of 46 beats per minute (bpm), with the heel lifted at the sound of the metronome and dropped on the next beat. The posture was maintained during the awaited time. A heel rise was counted if the back was straight, the foot passed the preset level of at least 5 cm, and the knee was fully extended. The muscle was judged to be fatigued based on two criteria: (1) the foot failed to reach the string, and (2) the pace could no longer be maintained for three consecutive repetitions. The tests were conducted by the same examiner using standardized instructions and verbal encouragements to each participant.

#### Experimental procedure

The experiment was conducted at National Cheng Kung University's Motion Analysis Laboratory from February to July 2020. The participants wore sport clothes with bare feet during the experiment. The anthropometric parameters, including the age, gender, height, and weight, were collected after video instruction of the experimental procedure was given, and each participant was asked to sign committee-approved informed consent. The participants then performed a brief warm-up for 5 min and familiarized themselves with the maximal voluntary isometric flexion (MVIC) and lunge forward movement tasks based on the received instructions.

**Figure 4:**
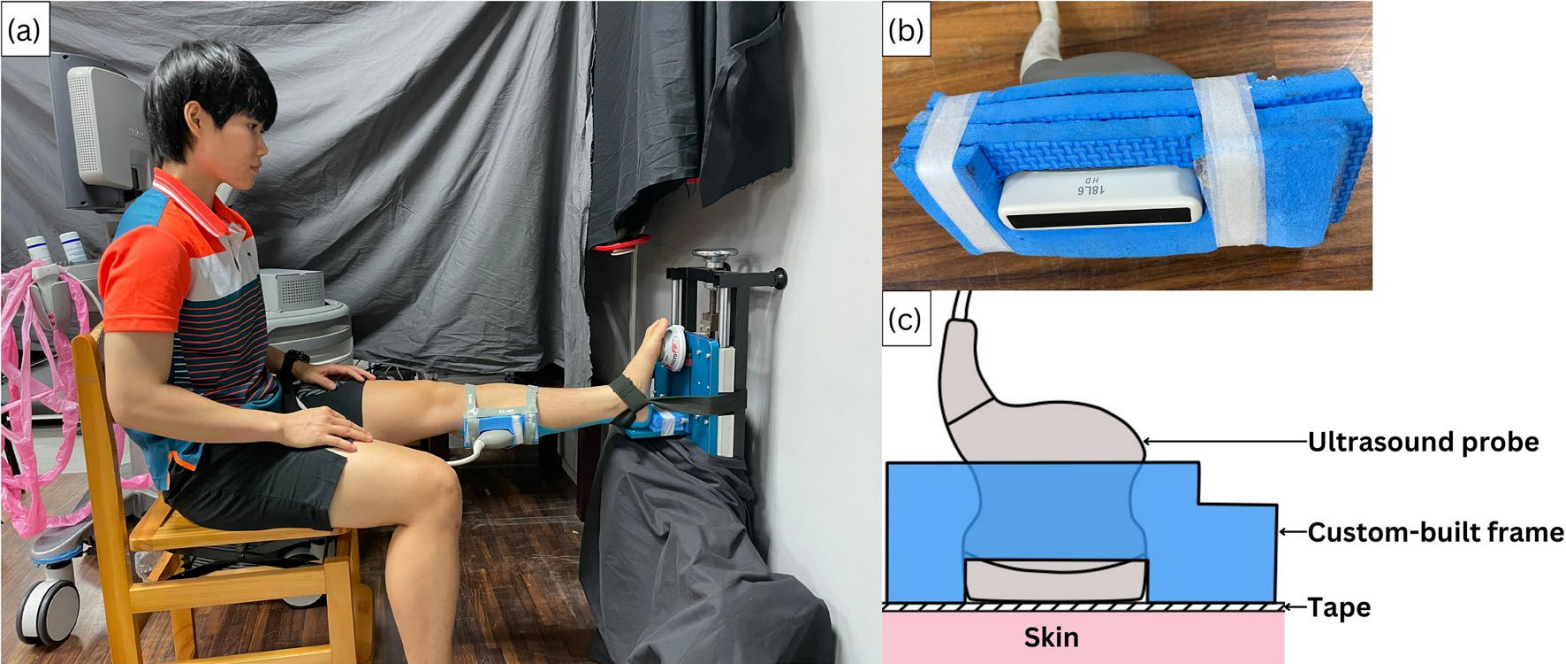
Maximal voluntary isometric plantar flexion task (**a**), the custom-built frame (**b**) and the cross-section view at the intersection of ultrasound probe and the tape (**c**).

In the MVIC task, the subjects were first applied taping on the posterior aspect of the leg which bordered the region bordering medial and lateral gastrocnemius boundary if it was the intervention sessions and then required to sit up straight with straps fastened across the foot and immobilization system and the knee fully extended, as shown in Fig. 4a. The plantar flexion force data were collected by a MicroFET2 Dynamometer (Hoggan Scientific, US) connected wirelessly to a laptop computer. For each participant, the force gage was adjusted such that it was at the same height as the medial cuneiform. Each subject was requested to perform plantar flexion for 5 seven-second trials. For each trial, the dynamometer was reset and the subject was then asked to steadily increase the force for three seconds and to hold this force for one second before relaxing. A time clock was provided to enable the subjects to monitor the procedure under the researcher's supervision.

In the lunge task, the subjects started with the prepared posture in badminton, namely the right leg placed in front of the left leg (for right-hand players). The lunge task comprised two phases, namely an eccentric phase and a concentric phase^27^. In the eccentric phase, the subject gradually transferred their weight onto the hindleg, leading to an increase of the dorsal ankle angle. On reaching the maximum dorsal angle (the end of the eccentric phase; Fig. 7 milestone 2), the participant performed ankle plantar flexion to drive the body forward. The concentric phase was judged to be complete when the hindleg foot left the ground.

**Figure 5:**
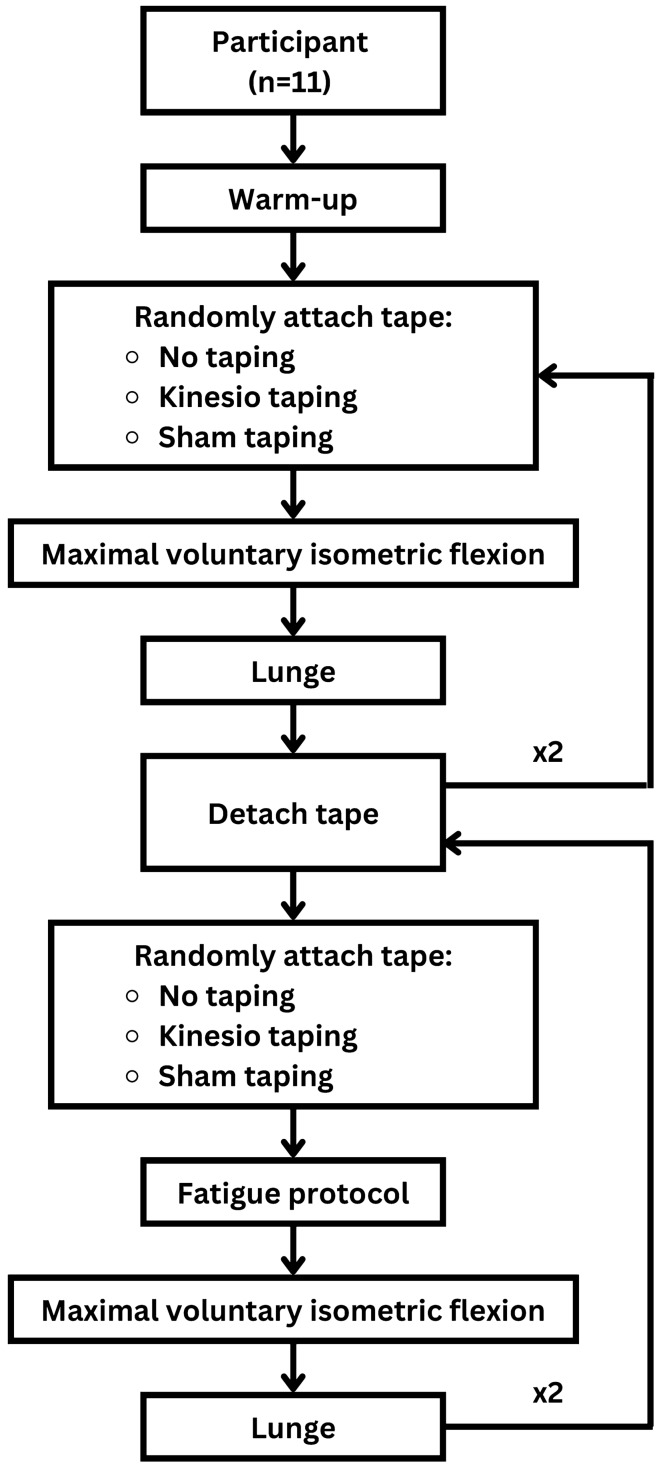
Schematic presentation of experimental procedure.

The overall experimental framework including two tasks (MVIC and badminton lunge) performed in two conditions (pre-fatigue and post-fatigue) as illustrated in Fig. 5. For the pre-fatigued condition, the two tasks were repeated three times by each participant with no taping (NT), kinesio taping (KT) and sham taping (ST), respectively, in a random arrangement. Once completing both tasks, the used tape was detached and the new one was applied immediately. The two tasks were carried out right after a 15-min period of taping and equipment application. And for the post-fatigued condition, another random sequence of NT, KT and ST was used and participants required to meet the fatigue criteria before performing two tasks. (Note that, for each condition, the random taping sequence was generated prior to the experiment using http://www.randomization.com/).

### Data recording

The architecture of the medial gastrocnemius muscle was measured by an ultrasound Acuson S2000 system (Siemens Healthcare, Erlangen, Germany) using an 18L6 HD linear-array transducer (Siemens Medical Solutions) with a range frequency of 6–18 MHz. The probe was placed at the mid-edge medial gastrocnemius muscle belly, which was directly over the tape in the case of the two taping interventions (ST and KT). For all three interventions, the probe position was marked in advance in order to assure the proper placement of the transducer. In addition, a custom-built frame was used to secure the probe in place without applying any additional compression force to the monitored area during MVIC and lunge movement as illustrated in Fig. 4b,c. In order to segment the lunge task for further analysis, the ground reaction force (GRF) was measured by two force platforms (981B, Kistler Instrument Corporation, Amherst, NY, USA) at a sampling rate of 1000 Hz and an eight-camera ExperVision Motion Analysis System which captured 15 standard reflective markers with a diameter of 25 mm attached on the anatomical landmarks of the lower trunk following the modified Helen-Hayes marker set. After post-processing, OrthoTrak 6.6 software was used to get the ankle angle.

### Data analysis

#### Muscle architecture measurement

**Figure 6:**
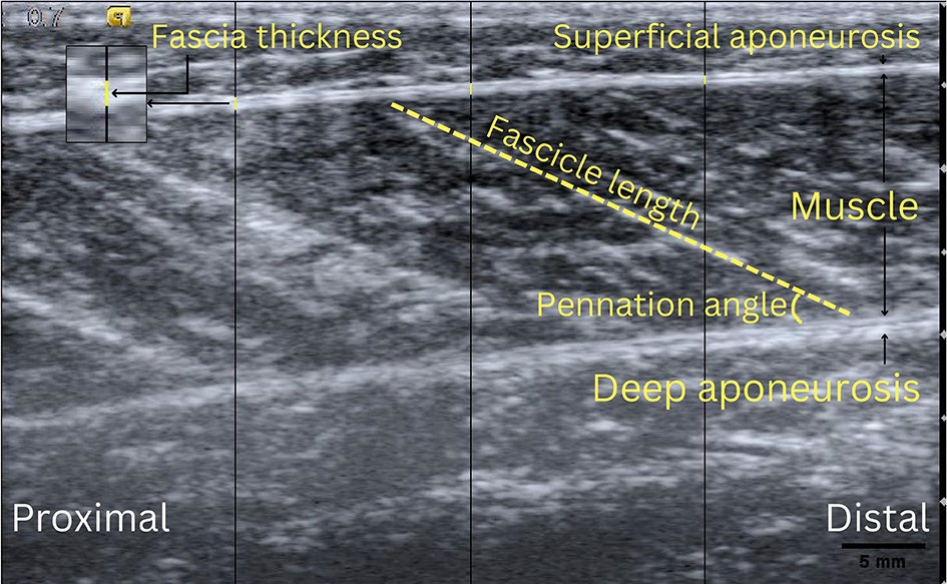
Measurement of fascia thickness, fascicle length and pennation angle.

For each subject, the muscle architecture was assessed by extracting the fascicle length, pennation angle and fascia thickness from the ultrasound video images acquired during task performance. The fascicle length (FL) and pennation angle (PA) were identified using the Simple Muscle Architecture Analysis (SMA) macro developed by Seynnes et al. in ImageJ software^28^. The macro defines the PA as the angle between the deeper aponeurosis and fascicle orientations, and the FL as the length of a straight line between the two aponeuroses. The fascia thickness (FT) was defined as the average value of the thickness measured at three equidistant locations (yellow straight-line mark), as shown in Fig. 6. All of the analyses were conducted by the same researcher. The intraclass correlation coefficient (ICC3, k: 0.669, 0.373–0.806) indicated fair reliability. Moreover, the FT measurement method was based on that described in a previous study with an intraclass correlation coefficient (ICC) of 0.67–0.77 and inter-observer coefficient (ICC) of 0.82–0.92^29^. The measurement errors of the fascia tissues varied in the range of 3–5%^30^.

#### Maximum voluntary isometric contraction (MVIC)

The plantar flexion motion in the MVIC task was captured using a seven-second ultrasound sequence, where the first frame coincided with the relaxed state of the muscle and the frame corresponding to the maximum force was extracted for further analysis.

#### Badminton lunge movement

**Figure 7:**
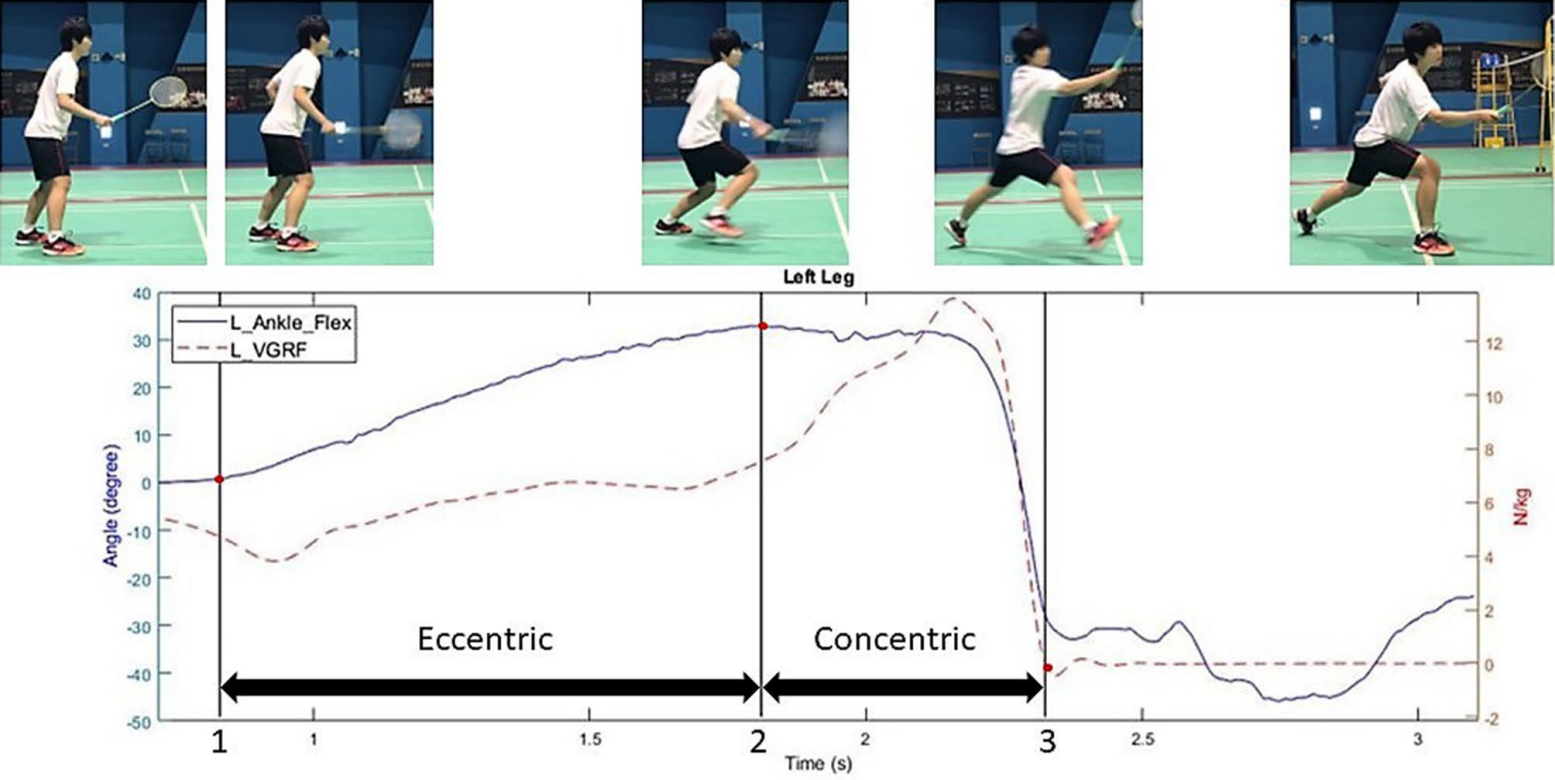
Data segmentation in lunge movement.

The lunge movement was divided into two phases (eccentric and concentric) based on three key milestones, namely the beginning of the eccentric phase, the transition point between the eccentric and concentric phases, and the end of the concentric phase, as identified from an inspection of the ankle angle and vertical ground reaction force data obtained by a motion camera system and force plate, respectively. As shown in Fig. 7, the beginning of the eccentric phase (1st vertical line) was taken as the start of left ankle dorsal flexion, while the transition point (2nd vertical line) was taken as the peak of the left ankle dorsal flexion, and the end of the concentric phase (3rd vertical line) was taken as the moment at which the vertical ground reaction force of the left leg dropped to zero.

### Statistical analysis

The mean and standard deviation (SD) values of descriptive statistics were used to express the results. The Friedman test was used to analyze parameter differences among the NT, KT and ST interventions. If there were significant differences, the significant pairwise comparison was found by using Wilcoxon signed-rank test for post-hoc analysis. The same test was also used to collate the fatigued condition with the non-fatigued condition in each taping intervention. The signal processing procedures were performed in MATLAB Software version R2018B, and all of the statistical analyses were conducted using PASW Statistics 26.0 (SPSS Inc., Chicago, IL, USA) with a statistical significance level of *p* < 0.05.

The sample size was determined by using G*Power 3.1.9.7 based on data of this study (n = 11) which compared the fascia thickness in no tape before and after fatigue protocol. After reaching the same size of n = 11, a priori power analysis was conducted with the effect size was 0.875 (Mean of difference was 0.07 and standard deviation of difference was 0.08). With a significance criterion of alpha was 0.05 and power was 0.80, the minimum sample size required with this effect size was n = 7 for Wilcoxon signed-rank test (matched pairs) and the effect size was 0.64 which considered to be large using Cohen’s (1988) criteria^31^. Therefore, the sample size of n = 11 is adequate to test the study hypothesis.

### Ethics approval

Human Research Ethics Review of National Cheng Kung University Committee approved this study. All participants gave written informed consent before data collection began.

### Consent to participate

Informed consent was obtained from all individual participants included in the study.

## Results

Eleven subjects were enrolled in the experiment. Each participant performed two tasks (MVIC and badminton lunge) under three taping interventions (NT, KT and ST) and two fatigue conditions (pre-fatigue and fatigued). As shown in Table 1, the peak isometric plantar flexion force in the NT, KT and ST groups following calf muscle fatigue induction declined significantly (NT, Z = − 2.934, *p* = 0.003; KT, Z = − 2.845, *p* = 0.004; and ST, Z = − 2.312, *p* = 0.021). However, there is no significance found among NT, KT and ST in both pre-fatigue and post-fatigue session (χ^2^(2) = 3.455, *p* = 0.178 and χ^2^(2) = 0.545, *p* = 0.761, respectively). The box whisker plot of the maximum isometric plantar flexion force was provided in the Supplementary Fig. S1 online.

**Table 1:** Maximum isometric plantar flexion force.

Variable	Tasks		NT	KT	ST	Friedman
			(n = 11)	(n = 11)	(n = 11)	*p *value
Force (N)	MVIC	Pre-fatigue	484 ± 111	458 ± 127	464 ± 106	0.178
		Post-fatigue	402 ± 80*	402 ± 96*	415 ± 101*	0.761

Data are presented as means + SD.

*NT* No taping; *KT* Kinesio taping; *ST* Sham taping; *SD* Standard deviation; *MVIC* Maximum voluntary isometric contraction.

*Statistically significant difference pre- and post-fatigue (Wilcoxon signed-rank test, *p* < 0.05).

The fascia thickness (FT) in the MVIC task showed a significant reduction in all groups (at rest: NT, Z = − 2.09, *p* = 0.036; KT, Z = − 2.20, *p* = 0.028; and ST, Z = − 2.24, *p* = 0.025; at peak force: NT, Z = − 1.79, *p* = 0.074; KT, Z = − 2.10, *p* = 0.036; and ST, Z = − 2.73, *p* = 0.006) (see Table 2). The results of the Friedman Test indicated that there was a statistically significant difference in FT at peak force flowing fatigue across the three interventions (NT, KT, ST, χ^2^(2) = 11.21, *p* = 0.004). The post-hoc test results showed that the FT of the KT group was significantly higher than that of the ST group (Z = − 2.81, *p* = 0.005). Among the three groups, only the NT group showed a significant (but slight) reduction in the FT at the beginning of the eccentric phase and at the end of the concentric phase of the lunge task (Z = − 1.74, *p* = 0.083 and Z = − 1.75, *p* = 0.081, respectively). Surprisingly, the Friedman Test (χ^2^(2) = 6.29, *p* = 0.043) resulted a light thicker FT in NT group than in the KT group at the transition stage between the eccentric and concentric phases of the lunge task in the pre-fatigue condition with post hoc analysis (Z = − 1.92, *p* = 0.055). The box whisker plot of the fascia thickness in MVIC and lunge tasks can be found in the Supplementary Fig. S2 online.

**Table 2:** Fascia thickness of medial gastrocnemius muscle.

Variable	Tasks		NT	KT	ST	Friedman
			(n = 11)	(n = 11)	(n = 11)	*p *value
FT (mm)	MVIC	At rest				
		Pre-fatigue	0.59 ± 0.08	0.58 ± 0.08	0.55 ± 0.07	0.120
		Post-fatigue	0.52 ± 0.09*	0.52 ± 0.06*	0.51 ± 0.07*	0.226
		At peak force				
		Pre-fatigue	0.56 ± 0.08	0.58 ± 0.06	0.55 ± 0.07	0.178
		Post-fatigue	0.52 ± 0.08	0.54 ± 0.05*^a^	0.49 ± 0.08*	0.004^†^
	Lunge	At the beginning of eccentric phase				
		Pre-fatigue	0.58 ± 0.08	0.54 ± 0.08	0.55 ± 0.08	0.275
		Post-fatigue	0.54 ± 0.06	0.55 ± 0.07	0.54 ± 0.07	0.643
		At the transition between eccentric phase and concentric phase				
		Pre-fatigue	0.60 ± 0.09	0.56 ± 0.08	0.57 ± 0.08	0.043^†^
		Post-fatigue	0.56 ± 0.06	0.56 ± 0.07	0.55 ± 0.06	0.513
		At the end of concentric phase				
		Pre-fatigue	0.60 ± 0.08	0.57 ± 0.07	0.56 ± 0.09	0.211
		Post-fatigue	0.56 ± 0.08	0.56 ± 0.06	0.54 ± 0.07	0.614

Data are presented as means + SD.

*NT* No taping; *KT* Kinesio taping; *ST* Sham taping; *SD* Standard deviation; *MVIC* Maximum voluntary isometric contraction.

*Statistically significant difference pre- and post-fatigue (Wilcoxon signed-rank test, *p* < 0.05).

^†^Statistically significant difference between NT, KT and ST groups (Friedman test, *p* < 0.05).

^a^Statistically significant difference to the ST group (Wilcoxon signed-rank test, *p* < 0.05).

The pennation angle (PA) of the medial gastrocnemius muscle peak force in the MVIC task before fatigue induction was significant different among three interventions (NT, KT, ST, χ^2^(2) = 6.73, *p* = 0.035) (see Table 3). Post hoc analysis with Wilcoxon signed-rank tests was conducted resulting that the PA was significantly lower in the ST group than in the NT (Z = − 2.13, *p* = 0.033) and KT (Z = − 2.40, *p* = 0.016) group. In addition, the fatigue protocol resulted in a significant reduction in the PA in the NT group at rest (Z = − 2.05, *p* = 0.041), in the KT group at the end of the concentric phase (Z = − 2.40, *p* = 0.016), and in the ST group at the beginning of the eccentric phase (Z = − 2.67, *p* = 0.008). The box whisker plot of the pennation angle in MVIC and lunge tasks can be found in the Supplementary Fig. S3 online.

**Table 3:** Pennation angle of medial gastrocnemius muscle.

Variable	Tasks		NT	KT	ST	Friedman
			(n = 11)	(n = 11)	(n = 11)	*p* value
PA (degree)	MVIC	At rest				
		Pre-fatigue	16.14 ± 3.30	16.72 ± 2.82	15.99 ± 2.75	0.913
		Post-fatigue	17.72 ± 2.17*	16.31 ± 2.90	17.31 ± 2.05	0.148
		At peak force				
		Pre-fatigue	22.44 ± 4.37^a^	22.65 ± 6.16^a^	19.88 ± 4.51	0.035^†^
		Post-fatigue	21.72 ± 4.21	21.77 ± 5.00	22.60 ± 6.94	0.148
	Lunge	At the beginning of eccentric phase				
		Pre-fatigue	15.56 ± 3.53	14.45 ± 2.99	14.20 ± 3.36	0.441
		Post-fatigue	16.22 ± 4.33	15.23 ± 3.91	15.86 ± 5.15*	0.307
		At the transition between eccentric phase and concentric phase				
		Pre-fatigue	23.57 ± 8.50	23.14 ± 7.07	23.74 ± 7.91	0.336
		Post-fatigue	23.19 ± 7.56	23.20 ± 8.43	24.28 ± 6.98	0.441
		At the end of concentric phase				
		Pre-fatigue	26.43 ± 7.86	25.79 ± 5.82	26.39 ± 6.89	0.913
		Post-fatigue	25.43 ± 8.19	23.16 ± 5.22*	23.77 ± 5.66	0.761

Data are presented as means + SD.

NT, no taping; KT, kinesio taping; ST, sham taping; SD, standard deviation; MVIC, maximum voluntary isometric contraction.

*Statistically significant difference pre- and post-fatigue (Wilcoxon signed-rank test, *p* < 0.05).

^†^Statistically significant difference between NT, KT and ST groups (Friedman test, *p* < 0.05).

^a^Statistically significant difference to the ST group (Wilcoxon signed-rank test, *p* < 0.05).

Finally, the fascicle length (FL) of the medial gastrocnemius muscle (see Table 4) in the post-fatigue condition showed a significant difference among the three groups at the transition point between the eccentric phase and concentric phase of the lunge task (χ^2^(2) = 7.09, *p* = 0.029). Following with the post hoc analysis, the FL of the KT group was notably longer than that of the ST group (Z = − 2.13, *p* = 0.033). The FL of the medial gastrocnemius muscle at the end of the concentric phase increased significantly following fatigue induction in all three groups (NT, Z = − 2.40, *p* = 0.016; KT, Z = − 2.67, *p* = 0.008; and ST, Z = − 2.13, *p* = 0.033). The box whisker plot of the fascicle length in MVIC and lunge tasks can be found in the Supplementary Fig. S4 online.

**Table 4:** Fascicle length of medial gastrocnemius muscle.

Variable	Tasks		NT	KT	ST	Friedman
			(n = 11)	(n = 11)	(n = 11)	*p* value
FL (mm)	MVIC	At rest				
		Pre-fatigue	69.60 ± 21.11	66.18 ± 15.91	66.57 ± 8.83	0.695
		Post-fatigue	62.46 ± 7.06	66.71 ± 14.49	64.15 ± 5.25	0.307
		At peak force				
		Pre-fatigue	48.24 ± 11.16	49.00 ± 13.05	54.99 ± 12.28	0.078
		Post-fatigue	50.81 ± 8.72	49.90 ± 10.99	49.93 ± 11.28	0.178
	Lunge	At the beginning of eccentric phase				
		Pre-fatigue	67.67 ± 14.15	70.01 ± 13.90	69.56 ± 11.47	0.913
		Post-fatigue	66.52 ± 14.14	70.20 ± 17.96	66.66 ± 14.11	0.529
		At the transition between eccentric phase and concentric phase				
		Pre-fatigue	46.33 ± 14.86	46.45 ± 15.64	46.16 ± 15.33	0.695
		Post-fatigue	48.76 ± 14.74	50.68 ± 18.24^a^	45.42 ± 13.88	0.029^†^
		At the end of concentric phase				
		Pre-fatigue	42.79 ± 14.62	43.39 ± 12.76	41.32 ± 12.74	0.761
		Post-fatigue	48.37 ± 14.56*	50.62 ± 15.20*	48.61 ± 15.77*	0.336

Data are presented as means + SD.

*NT* No taping; *KT* Kinesio taping; *ST* Sham taping; *SD* Standard deviation; *MVIC* Maximum voluntary isometric contraction.

*Statistically significant difference pre- and post-fatigue (Wilcoxon signed-rank test, *p* < 0.05).

^†^Statistically significant difference between NT, KT and ST groups (Friedman test, *p* < 0.05).

^a^Statistically significant difference to the ST group (Wilcoxon signed-rank test, *p* < 0.05).

## Discussion

The aim of this study was to determine the underlying mechanism of kinesio tape (KT) on the architecture of the medial gastrocnemius muscle beneath the tape following fatigue induction at the very instant moments of maximal voluntary isometric contraction (MVIC) and badminton lunge tasks. For reference purposes, measurements were also obtained using sham taping (ST), in which the wave-pattern elastic feature of KT was absent. No comparison was made between gender groups since previous studies have shown that the considered fatigue protocol (maximum standing heel rise) results in no notable differences between genders^32^. For both tasks, ultrasound images were used to examine the effects of KT on the real-time muscle architecture and fascia thickness beneath the KT.

In general, the results obtained in this study showed that the FT was significantly affected by fatigue in isometric contraction. In previous ultrasound studies, the plantar fascia thickness showed a slight reduction after running and walking tasks^24^. This may be the result of muscle tightening and thickening following fatigue, which compresses the fascia continuum and causes it to become thinner^33^. This may also account for the dramatic reduction in the FT of the post-fatigue gastrocnemius muscle at rest and at peak force during the present MVIC task for all three groups. A notable post-fatigue reduction of the FT also occurred in the NT group in the lunge performance task. However, for the KT group, the fascia thickness increased by 0.01 mm at the beginning of the eccentric phase, remained constant during the transition between the eccentric phase and concentric phase, and then decreased by 0.01 mm in the concentric phase. In other words, as hypothesized, the KT helped to shield the FT from fatigue effects during movement. Hence, it would create a space for lymphatic flow and enhance its velocity which was proved when taping the ankle of rabbit hind limb during passive movement^10^. As for the NT group, the fascia thickness in the ST group also reduced following fatigue, albeit to a lesser extent. Notably, the FT reduced (rather than increased, as in the KT group) since, in contrast to KT, the recoil effect in the ST took place in multiple directions rather than in the longitudinal stretching direction only. At the transition point between the eccentric and concentric phases, significant differences in the FT were observed among the NT, KT and ST groups (χ^2^(2) = 6.29, *p* = 0.043). Moreover, the FT of the NT group was slightly greater than that of the KT group (Z = 0.055, *p* = 0.055). In badminton lunges, the transition point between the two phases coincides with the moment at which the gastrocnemius muscle and taping are both in their most elongated condition. In the absence of taping, the skin is freely lifted by the bulked-up muscle after fatigue induction. By contrast, in the KT intervention, the dorsiflexion angle of the ankle reaches its maximum value at the transition point between the two phases, and hence the retraction force exerted by the tape also reaches its maximum value and compresses the skin area between the two anchors of the tape accordingly. Several studies have examined the effects of KT on superficial tissue deformation in the context of the tibia muscle^20^ and lower back skin^22^, respectively. Pamuk and Yucesoy^20^ examined the effects of KT taping on the tibia muscle and found a skin lifting effect on the medial side, but a skin compression effect on the lateral side. Cimino et al.^22^ found that the superficial skin layer was thinner at either end of the tape, but thicker along the lateral edges. And the measurements in^22^ were all conducted at the edges of the KT rather than under the tape, as in the present study. However, neither study considered the architecture of muscle in prompting a stretching or retraction movement of the KT. In general, the present results suggest that the effectiveness of KT can be attributed to prevent a reduction in the FT due to fatigue, which allows more space for the fascia to be lifted. Notably, the lunge movement results in a greater KT tension effect than the isometric contraction movement, in which the ankle angle is fixed and the tension force applied by the KT remains constant. Furthermore, through repeated stretching and retraction, the KT mimics manipulative therapy, which increases the thickness of the thoracolumbar fascia and retains this effect for 24 h thereafter^34^. In conclusion, the effect of KT on the FT is more effective in movement than in isometric contraction.

The fascia layer is an intramuscular extracellular matrix structure consisting of epimysium and endomysium tissue surrounding muscle fiber^8,35^. In the present study, it is hypothesized that if the force applied by the KT is able to reach these tissue layers, it may cause a change in the muscle architecture, such as the pennation angle (PA) and fascicle length (FL). Previous studies have reported that the muscle architecture is diversely affected depending on the fatigue protocol^36–39^. For example, stretch–shortening cycle exercise lessens the PA and lengthens the FL^39^, whereas other exercises, such as sustained submaximal isometric plantar flexion and repeated isometric plantar flexion maximal voluntary contraction, lead to a growth in the PA and a reduction in the FL^36,38^. The present heel-rise fatigue protocol led to a significant increase of the PA at rest (Z = − 2.05, *p* = 0.041) for the NT group in the MVIC task, while the FL reduced non-significantly (Z = − 0.98, *p* = 0.328). Under the circumstances of no fatigued effect, the PA at peak force in the MVIC task was significantly lower in the ST group than in the NT and KT groups (Z = − 2.13, *p* = 0.033 and Z = − 2.40, *p* = 0.016, respectively) due to the effects of the ST in compressing the skin over the muscle. By contrast, the absence of tension in the NT group and the wave pattern of the kinesio tape in the KT group facilitated further stretching. Notably, the present results conflict with the findings of Obst et al.^40^ that deloading tape lessens fascicle shortening during ankle plantar flexion^40^. However, in Obst et al.^40^, the KT was applied in a diamond shape rather than a Y shape, as in the present study, and the ultrasound transducer was positioned above the taped skin. During the lunge task, the PA showed a significant increase (Z = − 2.67, *p* = 0.008) after fatigue induction in the ST group at the beginning of the eccentric phase and a significant reduction (Z = − 2.40, *p* = 0.016) after fatigue induction in the KT group at the end of the concentric phase. By contrast, the FL between pre- and post-fatigued condition increased (Z = − 1.87, *p* = 0.062) in the KT group at the transition between the eccentric and concentric phases in the fatigue condition. This can be attributed most likely to a compression of the KT due to a bulking up of the underlying muscle as a result of fatigue. The pressure generated from the combination of KT and muscle movement is similar in this regard to the effects of compression garments, which gradually distribute the enhancement of blood circulation from the distal to proximal regions^41^. In general, compression garments lead to a reduction in the vein diameter by the aligned valves and hence drives the venous blood back to the heart^42^. The allocation of pressure is a key factor affecting blood circulation and, in the context of the present study, leads to the FL difference between the KT and ST groups since the wave pattern of the KT causes the stretching and recoil effect to take place longitudinally, whereas in the ST group, the tension of the tape stretches in various directions. Consequently, the FL of the KT group in the fatigue condition is longer than that in the ST group (Z = − 2.13, *p* = 0.033) because inhibited muscle activation may reduce active muscle force of the medial gastrocnemius^40^. Importantly, this benefits the performance of athletes since the FL is proportional to the muscle fiber velocity^43^, and a greater muscle fascicle length increases the plantar flexor torque^44^. Overall, the KT during movement generated an appropriate dynamic pressure variation and sufficient periodic compression and decompression of fascia layer. As a result, the magnitude of myofascial loads was distributed locally, thereby impairing the mechanics of activated muscles in a significant way^21,40^. The heel-rise fatigue protocol caused the FL to lengthen at the end of the concentric phase of the badminton lunge movement in all three groups (NT, KT and ST). However, previous studies have noted that the PA and FL are both strongly affected by the knee and ankle angle^37,39^. Thus, both factors should be controlled and further investigated in order to verify this claim.

Several limitations of this study should be acknowledged. In particular, the sample size was very small (n = 11), and hence care should be taken in generalizing the results to a larger population. Furthermore, the present study took no account of the effects of KT application on the biomechanical properties of the muscle, such as the muscle stiffness, under dynamic movement. However, previous studies have reported that the application of KT may not only affect the muscle morphology, but also alter the biomechanical properties of the muscle tissues^21,45^. The elastic properties of muscles strongly affect the tendon and muscle performance^46–48^. Accordingly, further studies on muscle properties are required in order to properly understand the mechanisms of KT.

Since Kinesio Taping Method was invented by Dr. Kase in 1973, it has been widely used by the athlete for the treatment and prevention of sport related injuries^5,6^. Thereafter, a study in rabbit hind leg proved the effect of KT in increase the lymphatic drainage in 2003^10^. Later in 2015, Magnetic resonance imaging was used to quantity the acute effect of KT on the local tissue deformation of the medial tibialis anterior during static prone position^20^. With the proof of local tissue deformations caused by KT method, Wang et al. used magnetic resonance elastography to find out a decrease in the shear stiffness of paraspinal muscles right after KT application in 2018^21^. While the KT often uses in daily life or sport, it is difficult for magnetic resonance imaging and elastography to observe a dynamic movement. Therefore, in 2018, Cimino et al. made an effort to detect a possible pathway for functional benefits of KT application by using ultrasound to observe all edges of KT during neutral, flex and extended spine posture^22^. Making use of the ultrasound to discover the area under the skin in real-time, Obst et al. in 2019 was able to examine the medial gastrocnemius muscle fascicle during heel rise exercise after applying the diamond shape taping around the ultrasound probe^40^. However, both studies using ultrasound only investigated the area near the tape which may not explain the real mechanism underneath KT.

This study is the first reported attempt in the literature to observe the muscle architecture and fascia thickness beneath KT during isometric contraction and dynamic movement. Although the results do not statistically confirm that KT thickens the fascia thickness after fatigue induction, no dramatic reduction in the fascia thickness was observed; whereas in the ST and NT interventions, a lessening of the fascia thickness occurred under both isometric contraction and lunge movement. In general, the results suggest that the benefits of KT are more pronounced for dynamic movement, such as badminton lunges, than for isometric contraction, in which the tape undergoes only very slight change in length during contraction. Future studies may usefully examine the mechanical properties of muscle and muscle activities during movement in order to further understand the basic mechanisms of KT.

## Supplementary Information

Supplementary Information.

## Data Availability

The datasets generated during and/or analysed during the current study are available from the corresponding author on reasonable request.
