# Supplementary material for: Behavior of medial gastrocnemius muscle beneath kinesio taping during isometric contraction and badminton lunge performance after fatigue induction
Source: Sci Rep. 2023 Jan 31;13:1779. doi: 10.1038/s41598-023-28818-3 (PMC9889375; doi:10.1038/s41598-023-28818-3)
Supplement: Supplementary file 1 — Supplementary Information. [file 41598_2023_28818_MOESM1_ESM.pdf]

Supplementary Figures

Title: Behavior of medial gastrocnemius muscle beneath kinesio taping during isometric contraction and badminton lunge performance after fatigue induction

Authors:

1. Minh Hoang-Thuc Vo, Department of Biomedical Engineering, National Cheng Kung University, Tainan, Taiwan, minhvo.janet@gmail.com
2. Chien-Ju Lin, Department of Biomedical Engineering, National Cheng Kung University, Tainan, Taiwan, sweetchien2@gmail.com
3. Hsiao-Feng Chieh, Department of Biomedical Engineering, National Cheng Kung University, Tainan, Taiwan, hfchieh@gmail.com
4. Li-Chieh Kuo, Department of Occupational Therapy, National Cheng Kung University, Tainan, Taiwan, jkkuo@mail.ncku.edu.tw
5. Kai-Nan An, Division of Orthopedic Research, Mayo Clinic, Rochester, U.S., an.kainan@mayo.edu
6. Yu-Lin Wang, Department of Rehabilitation, Chi Mei Medical Center, Tainan, Taiwan, d8101080@gmail.com
7. Fong-Chin Su, Department of Biomedical Engineering, National Cheng Kung University, Tainan, Taiwan, fcsu@mail.ncku.edu.tw

***Keywords*:** Kinesio tape; ultrasound; fascia thickness; muscle architecture; isometric contraction; badminton lunge; fatigue.

***Correspondence*:** Fong-Chin Su, Department of Biomedical Engineering, National Cheng Kung University, Tainan, Taiwan, fcsu@mail.ncku.edu.tw

***Number of Supplementary Figure*:** 4

Supplementary Figure S1 Box whisker plot of the maximum isometric plantar flexion force.

Supplementary Figure S2 Box whisker plot of the fascia thickness in MVIC and lunge tasks.

Supplementary Figure S3 Box whisker plot of the pennation angle in MVIC and lunge tasks.

Supplementary Figure S4 Box whisker plot of the fascicle length in MVIC and lunge tasks.

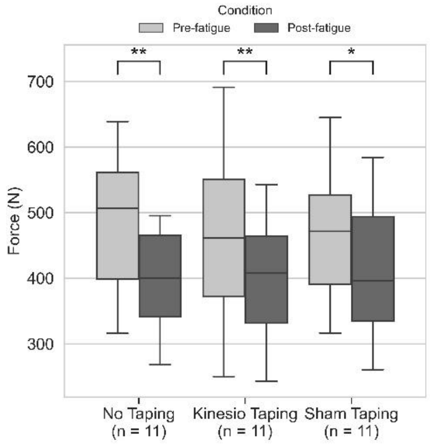

Fig. S1 Box whisker plot of the maximum isometric plantar flexion force. Statistically significant difference using Wilcoxon signed-rank test (*p < 0.05, **p<0.01).

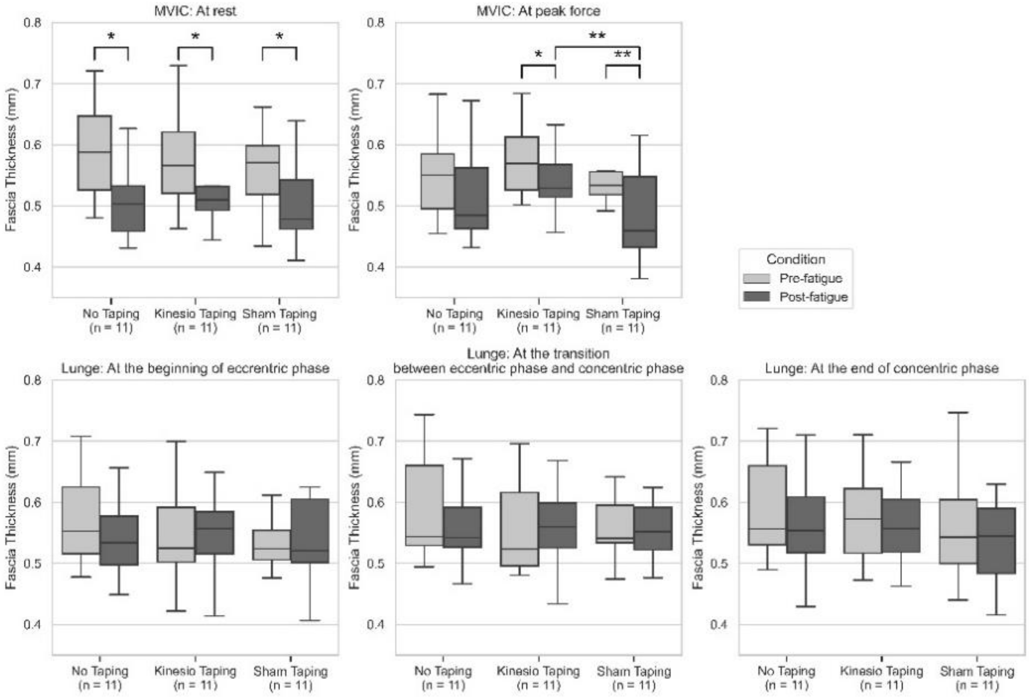

Fig. S2 Box whisker plot of the fascia thickness in MVIC and lunge tasks. Statistically significant difference using Wilcoxon signed-rank test (*p < 0.05, **p<0.01).

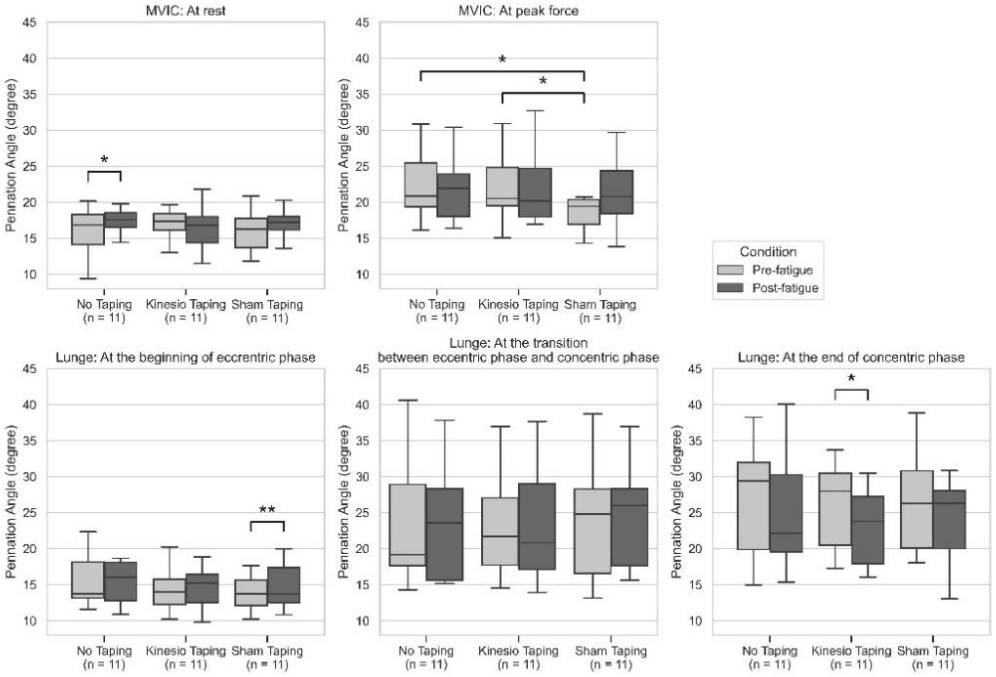

Fig. S3 Box whisker plot of the pennation angle in MVIC and lunge tasks. Statistically significant difference using Wilcoxon signed-rank test (*p < 0.05, **p<0.01).

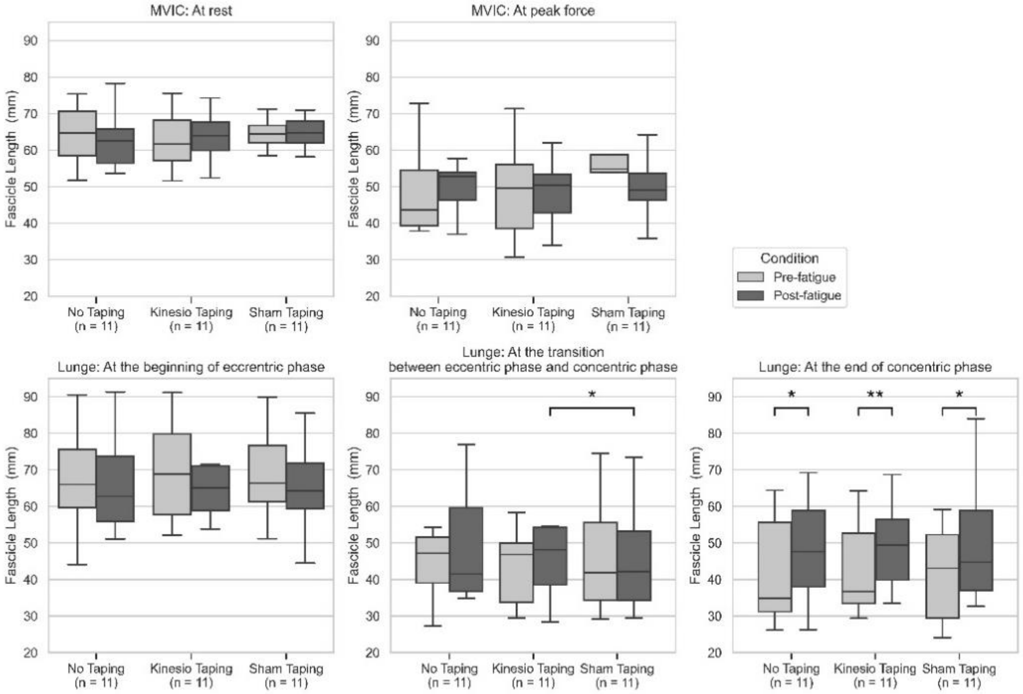

Fig. S4 Box whisker plot of the fascicle length in MVIC and lunge tasks. Statistically significant difference using Wilcoxon signed-rank test (*p < 0.05, **p<0.01).
